# Modification of cryoprotectants on sperm cryopreservation: A study of embryo development

**DOI:** 10.5455/javar.2024.k804

**Published:** 2024-08-26

**Authors:** Manggiasih Dwiayu Larasati, Silvia Werdhy Lestari, Mulyoto Pangestu, Andon Hestiantoro, Gito Wasian

**Affiliations:** 1 Doctoral Program in Biomedical Sciences, Faculty of Medicine, Universitas Indonesia, Jakarta, Indonesia; 2 Department of Medical Biology, Faculty of Medicine, Universitas Indonesia, Jakarta, Indonesia; 3 Education Program in Reproduction and Development, Department of Obstetrics and Gynecology, School of Clinical Sciences, Monash University, Melbourne, VIC, Australia; 4 Division of Reproductive Endocrinology and Infertility, Department of Obstetrics and Gynecology, Faculty of Medicine, Universitas Indonesia, Jakarta, Indonesia

**Keywords:** Cryoprotectant modification, embryo development, sperm cryopreservation

## Abstract

**Objective:**

This research was conducted to analyze the effect of cryopreservation of sperm using modified cryoprotectants on embryo development through *in vitro* fertilization (IVF). In this research, three types of cryoprotectant combinations were compared, including Nakagata, modified cryoprotectant, and commercial (Kitazato).

**Materials and Methods:**

Several parameters, namely sperm concentration, motility, morphology, plasma membrane integrity, cryo-survival rate, and viability rate, were measured and compared before vitrification and warming. Embryo development was also observed on the first and third days of development based on the cell number, cell size, and fragmentation rate.

**Results:**

Sperm cryopreservation exhibited a negative influence on embryo quality. Both Nakagata cryoprotectants and modified cryoprotectants attained good-quality embryos. In terms of embryonic development, this research revealed a modified cryoprotectant superior to Nakagata’s cryoprotectant, although Kitazato was superior to the Nakagata cryoprotectant and modified cryoprotectant. Significant differences were found in the three cryoprotectants on observations on day 1 and day 3, all of them with *p-*value < 0.05.

**Conclusion:**

Modified cryoprotectant was found to be better than Nakagata but less significant than Kitazato in terms of embryonic development quality. Therefore, modified cryoprotectants could be a better alternative compared to commercial (Kitazato) cryoprotectants for improving embryo quality.

## Introduction

Sperm cryopreservation has been widely used in assisted reproductive technology programs, including infertility treatment and fertility preservation. Sperm conservation also maintains the fertility of the sperm by storing them before chemotherapy, radiotherapy, or any surgery that could affect the reproductive system [1]. Cryopreservation is a process of preserving and storing animal, plant, and biological material cells by reducing metabolic activity without affecting the organelles in the cells at very low temperatures (−196°C) in liquid nitrogen. The biological and morphological functions of the cells are maintained optimally after thawing. However, such a process causes damage, even death or apoptosis, to the cells, both in the cell membranes and other organelles [2]. Besides that, sperm cryopreservation also involves extreme temperatures and causes cold shock. The main effect of cold shock on spermatozoa cells is a decrease in motility and vitality, as well as changes in component lipids in the spermatozoa membrane. Therefore, an optimal freezing temperature is required before the cells are stored in liquid nitrogen to avoid cryoinjury. Currently, several efforts have been developed to overcome cell injury due to cryopreservation, such as the use of seminal plasma microvesicles and exosomes [3] or the addition of 5% Platelet Rich Plasma (PRP), which significantly increases sperm progressive motility, viability, and membrane integrity after cryopreservation in normozoosperm [4].

The cryopreservation process requires cryoprotectants to maintain sperm function. Cryoprotectants are non-electrolyte chemicals that reduce the lethal effect during freezing, either in the form of a solution or the formation of ice crystals to maintain the quality of the cells [6]. In addition, during cell thawing, cryoprotectants must be removed to prevent toxicity [7]. In general, cryoprotectants are classified into permeable and non-permeable groups. Permeable or intracellular cryoprotectants can move in and out of the cell membrane as they have a small molecular size to replace the water in the cell, e.g., glycerol, dimethyl sulfoxide, dimethyl acetaldehyde, propylene glycol, and ethylene glycol [8]. Meanwhile, non-permeable or extracellular cryoprotectants cannot penetrate cell membranes due to their large molecules, yet they provide better protective agents against cell damage, such as raffinose, sucrose, egg yolk citrate, albumin, and polyethylene glycol [9].

Glycerol has been the most widely used cryoprotectant in mammalian sperm freezing. Experts have tested various freezing methods and cryoprotectants, where the combination of glycerol and liquid nitrogen remains the golden standard [10]. Glycerol slowly penetrates the cell membrane and balances cell conditions in the cytoplasm, reducing intracellular water volume without leading to cell dehydration [1]. Sztein’s research demonstrated the ability of 6% glycerol to fertilize 62% of egg cells during the cryopreservation of rat sperm [11].

Currently, Nakagata is a medium commonly used to store frozen mouse sperm [12]. Based on the Nakagata Protocol, the cryoprotectants comprise 18% raffinose and 3% skim milk. The fertility rate can be decreased from 70% to 26% to 13% [11]. Raffinose can increase the viscosity and lower the freezing point of extracellular fluids, even when cellular dehydration occurs quite immediately [1]. After thawing and elimination of cryoprotectants, the motility of mice sperm reaches 59% on raffinose [11].

Another commercial cryopreservation medium commonly used for the frozen storage of human sperm is called Kitazato. This medium uses glycerol and trehalose in the vitrification process. *In vitro* fertilization (IVF) for human sperm resulted in a survival rate of 60%. Kitazato can also increase fertilization in cryopreserved mice. After thawing, the sperm recovery rate reached 83% with a motility of 48% [13]. However, Kizatato is a relatively expensive procedure with major challenges in the translation process from Japan. Therefore, there is a need for an alternative to a modified cryoprotectant using a combination of glycerol and raffinose. However, sperm quality influences embryogenesis from a very early stage, where lower sperm quality leads to a lower fertility rate, cleavage rate, and blastocyst development [14]. In addition, damage to sperm deoxyribonucleic acid significantly reduces the fertility rate of embryos into blastocysts in mice [15]. However, many factors affect sperm quality in freezing and thawing, e.g., the freezing method, temperature control, sperm preparation technique, and type of cryopreservative agent. Likewise, many factors affect oocyte quality. Ideally, good-quality sperm and oocytes will produce good embryos too. On the contrary, the embryonic quality will decrease if the sperm quality is low due to frozen storage. Therefore, cryoprotectant modification needs to be further developed for a more affordable price (cost-effectiveness), easy availability, even a higher survival rate, and optimal sperm and embryo quality.

## Materials and Methods

### Ethical approval

This research has gained an ethical permit from the Ethics Committee of the Faculty of Medicine, Universitas Indonesia, under the number KET-326/UN2.F1/ETIK/PPM.00.02/2022.

### Criteria for experimental animals

Male *Musculus albinus* strain Deutchland Denken Yoken (DDY) mice aged 12–15 weeks and females aged 8–10 weeks were obtained from the Faculty of Animal Husbandry, Bogor Agricultural University, Indonesia, as the experimental animals. Mice were acclimatized at Animal Research Facilities - Institute of Medical Education and Research Indonesia. Mice were fed and consumed water *ad libitum*. The treatment and handling of experimental animals were carried out based on the guidelines for animal trials.

In this study, male mice were put into four groups: Treatment Group 1 (Nakagata Protocol), Treatment Group 2 (Modified Cryoptrotectant Method), Treatment Group 3 (Kitazato Cryoprotectant), and Control Group (Fresh Sperm). Based on Federer’s formula, the number of male mice per group was set to 16, totaling 64 mice selected out of 70 mice. Six mice were not selected due to failure to retrieve sperm from the epididymis, and five female mice were also excluded due to a lack of response to ovarian stimulation.

### Sperm collection and preparation

Euthanasia with cervical dislocation had been performed before taking out the cauda epididymis. The cauda epididymis was placed in 200 μl of sperm rinse (Vitrolife Cat. No. 10101, Sweden) in an Eppendorf tube (Biologix Cat. No. 80-0015, USA). An incision was performed in each cauda epididymis to let the sperm cell out of the epididymis and swim to the surface of the medium. Sperm preparation was done using the simple washing method. Samples and medium were put into a tube of the same size and centrifuged at 1800×g for 10 min. The supernatant was discarded, and then the pellet was suspended in 100 μl of G-IVF™ PLUS (Vitrolife Cat No. 10134, Sweden). The sperm solution was placed in a freezing tube and floated above liquid nitrogen in a cryobiological container.

### Sperm cryopreservation

Sperm cryopreservation was performed using the vitrification method with different cryoprotectants (Nakagata, Modification, or Kitazato methods).

**Cryopreservation**

Nakagata Method: Sperm cryopreservation using the Nakagata Protocol was performed by dissolving 3.6 gm of raffinose (Sigma Aldrich Cat. No. 83400) into 16 ml of distilled water at 60°C. Raffinose was then dissolved until it formed a clear solution, and then 0.6 gm of skim milk (Sigma-Aldrich Cat No. 70116) was added and distilled with water until the volume reached 20 ml [12]. One point five ml of the solution was put in tubes and centrifuged at 10,000×g for 15 min at room temperature. The supernatant was put in a new tube to be filtered using a disposable filter. The cryoprotectant was then put into the tubes and stored at 4°C.

Modification method: It was conducted by dissolving 3.6 gm of raffinose in 15 ml of distilled water at 60°C before adding 0.6 gm of skim milk, then adding 2 ml of glycerol (Sigma Aldrich Cat. No.C6039), and redistilling it again until the volume reaches 20 ml. One point five ml of the solution was put into tubes and centrifuged at 10,000×g for 15 min at room temperature. Finally, the cryoprotectant was put into the tubes to be stored at 4°C.

The Kitazato Method. The sperm sample was added to SpermFreeze SF3-10 cryoprotectant (Kitazato Biopharma Company Ltd., Japan, Cat. No. 92211) in a ratio of 1:1. Then, 200 μl of each sample was put into a sterile polyethylene terephthalate glikol (PETG) sperm straw (CBS-IMV Cat. No. 018917, USA) and sealed at both ends of the straw. It was then immediately transferred to a storage tank.

**Warming**

The straws were briefly immersed for 10 sec in a 37°C water bath, and then transferred into tubes. Subsequently, 10 μl of thawed sperm was added to 100 μl of EmbryoMax^®^ human tubal fluid (HTF) medium (Sigma-Aldrich Cat No. MR-070-D). The mixture was placed in a tube and stored in an incubator at 37°C for 15 min. Afterward, aliquots of post-thawing sperm were utilized for either fertilization or microscopic examination.

### Sperm quality

The parameters of sperm quality observed in this study were concentration, motility, Cryosurvival Rate (CSR), morphology, viability, Viability Rate (VR), and integrity of the plasma membrane. Sperm concentration and motility were counted using a Makler counting chamber (Sefi-Medical Instruments, Haifa, Israel) by adding a 10 μl sperm sample into the chamber. Furthermore, each 10 μl sperm sample was pipetted onto a glass slide to examine motility, morphology, and viability. Likewise, the cryosurvival rate was defined and determined based on the total motility index before and after cryopreservation. The viability rate was determined by analyzing each group both before freezing and after thawing for each subject within that group. The integrity of the plasma membrane in this study was tested using the hypoosmotic swelling test (HOST) by mixing 50 μl of sperm sample with 1 ml of hypo-osmotic solution. At least 100 sperm are analyzed for each sperm quality parameter.

### Oocyte collection

The superovulation of female mice was achieved through the intraperitoneal injection of 15 IU of pregnant mare serum gonadotropin (PMSG) (Sigma Aldrich Cat. No. G4877) and 15 IU of human chorionic gonadotropin (hCG) (Sigma Aldrich Cat. No. CG10). Subsequently, euthanasia was carried out through cervical dislocation, occurring approximately fifteen to seventeen hours post-hCG injection. Following euthanasia, the oviducts were carefully removed [16]. These oviducts were then transferred into G-MOPS™ PLUS (Vitrolife Cat No. 10130, Sweden) at an appropriately warmed temperature. From the ampulla of the oviducts, cumulus oocyte complex (COC) was collected and subsequently placed into a fertilization medium for further processing.

### IVF

Medium and dishes were prepared the day before the IVF. 50 μl G-IVF™ PLUS medium (VitrolifeCat No.10134, Sweden) or 30 μl of each G-1™ PLUS (Vitrolife Cat No. 10128, Sweden) were placed in the fertilization or culture dish, each covered with OVOIL™ (Vitrolife Cat No. 10029, Sweden). Both dishes were incubated overnight in an incubator at 37°C with 5% CO_2_. The fertilization process is carried out using conventional IVF by adding into each drop containing COC at a concentration of 250 thousand sperm cells/ml, under a controlled gas mixture comprising 5% CO_2_. After 4–6 h of incubation, the zygote was aspirated and transferred to the culture dish of G-1™ PLUS medium. The IVF groups were P1: cIVF using sperm cryopreservation using Nagakata Protocol and fresh oocytes; P2: cIVF using cryoprotectant modification and fresh oocytes; P3: cIVF using Kitazato and fresh oocytes; and C the control group: cIVF using fresh sperm and oocytes.

### Observation of embryo development

The results of IVF were observed on the first and third days using an inverted microscope, and the quality was analyzed using Gardner’s modification [17]. Cleavage-stage embryos were morphologically graded according to blastomere regularity, degree of fragmentation, and cell number. The embryos were then classified into good, moderate, and poor categories. Based on the similarity in size, good-quality embryos were those with more than 50% of blastomeres of the same size, in the moderate category (30%–40%) and in the poor category (10%–20%). Based on the degree of fragmentation, good-quality embryos had less than 25% fragmentation, moderate (10%–25%), and poor (<25%). Embryos were also classified based on the number of blastomeres; a good-quality embryo developed two cells in the cleavage stage on the first day and 7–9 cells on the third day. Embryos of moderate quality formed two cells in the cleavage stage on the first day and 5–6 cells on the third day, while those of poor quality failed to divide their cells (only 1 cell) on the first day or less than five cells on the third day. In this study, the grading was simplified into two categories; where the good and moderate categories were put under the good category and the poor category remained. Embryos resulting from IVF were observed using an inverted microscope with 400× magnification.

### Statistic analysis

Research data were analyzed using SPSS version 22, IBM. Respectively, the data were analyzed using the t-test to find the differences between the two groups and then an ANOVA to find the differences between the three groups. The post hoc test was carried out using the LSD test. The difference is considered significant if the *p-*value ≤ 0.05.

## Results and Discussion

### The effect of cryoprotectant modification on sperm quality

Parameters of sperm quality and distribution frequency of each group are presented in Table 1, where the mean value of sperm concentration of each treatment group decreased after sperm cryopreservation in comparison to the fresh sample group (control). In this study, three different types of cryoprotectants were used, namely Nakagata (raffinose and skim milk), Modification (glycerol, raffinose, and skim milk), and Kitazato (trehalose and glycerol). All cryoprotectant kits were combined, resulting in good sperm qualities. Likewise, Borini et al. [4] also found the combination of cryoprotectants better than a single cryoprotectant.

Cryoprotectants were regarded as good when the sperm concentration remained or slightly changed. A significant difference in sperm concentration before and after vitrification was found in the Nagakata groups. In addition, Kitazato was not significantly better than Modified. In the experiment, Kitazato appeared to be the best treatment. Sperm concentration is a relatively unreliable parameter of semen since it is highly affected by the dilution process [18]. On the other hand, concentration is an important parameter in the handling of semen for use or preservation [19]. Meanwhile, sperm cryopreservation with minimal concentration also affects post-thawing sperm quality, especially motility [20].

Similar to the sperm concentration parameter, there was a significant difference in the sperm motility parameter between before and after vitrification in the Nagakata and modified groups. Darsini [21] also found lower motility after sperm cryopreservation. This study confirmed that cryopreservation is often associated with decreased sperm motility and fertilizing potential due to damage to the sperm plasma membrane and acrosome [22]. The results showed that the addition of raffinose to the modified cryoprotectant medium positively affects the percentage of motile and viable sperm in the epididymal sperm of mice after thawing. This can be used as an indicator that raffinose as a type of sugar is effective in protecting sperm from damage during the cooling, freezing, and thawing processes to improve sperm quality.

There was a significant difference before and after vitrification in terms of sperm viability for all groups: the Nakagata, Modified, and Kitazato groups. Cryoprotectants were considered good when they kept the level of sperm viability or only made a slight reduction to it. For this parameter, all groups showed insignificant differences, with Nakagata appearing to be the best, and the mean viability rate of the modified group was higher than Kitazato. Therefore, we confirmed that modification cryoprotectant could restore sperm motility ability after frozen storage by 36%, although it remains outperformed by Kitazato, which achieved a 43% improvement. The results of this study contradict previous studies, which stated that there was almost a 50% reduction in sperm motility after cryopreservation of sperm [23]. Although sperm quality parameters decreased, the results showed that sperm recovery was quite reliable in each treatment group.

**Table 1.** Sperm quality analysis, cryosurvival rate, and viability rate.

No	Parameter	Fresh (control)	Warming groups			*p-*value
			Nakagata	Modified	Kitazato	
		*n =* 48	*n =* 16	*n =* 16	*n =* 16	
1	Concentration (million/ml)	3.66 ± 0.23	1.89 ± 0.24	2.81 ± 0.22	3.10 ± 0.34	0.000^a)^ 0.007^e)^ 0.690^b)^ 0.488^f)^ 0.818^c)^ 0.000^g)^ 0.009^d)^
2	Motility (%)	48.06 ± 2.05	40.63 ± 3.81	36.19 ± 2.53	43.50 ± 2.10	0.014^a)^ 0.515^e)^ 0.000^b)^ 0.034^f)^ 0.009^c)^ 0.012^g)^ 0.341^d)^
3	Viability (%)	51.98 ± 2.24	45.00 ± 1.75	38.00 ± 3.67	36.50 ± 4.28	0.007^a)^ 0.081^e)^ 0.019^b)^ 0.792^f)^ 0.001^c)^ 0.000^ )^ 0.096^d)^
4	Morphology (%)	64.19 ± 1.68	33.19 ± 1.86	41.78 ± 2.37	41.25 ± 2.95	0.000^a)^ 0.029^e)^ 0.000^b)^ 0.948^f)^ 0.000^c)^ 0.000^g)^ 0.014^d)^
5	Sperm membrane integrity (%)	64.38 ± 1.29	40.25 ± 1.78	53.94 ± 2.19	55.88 ± 1.47	0.000^a)^ 0.000^e)^ 0.023^b)^ 0.470^f)^ 0.124^c)^ 0.000^g)^ 0.000^d)^
6	Cryosurvival Rate (%)	-	42.38 ± 4.74	54.08 ± 6.73	65.43 ± 7.29	0.168^d) ^0.266^f)^ 0.015^e)^ 0.049^g)^
7	Viability Rate (%)	72.13 ± 2.41	69.56 ± 3.92	65.44 ± 4.58	66.69 ± 3.61	0.582^a) ^0.594^e)^ 0.206^b) ^0.832^f)^ 0.220^c)^ 0.586^g)^ 0.499^d)^

*Note:* Data are presented as mean ± standard error mean (mean±SEM). ^a)^The fresh sample vs Nakagata (F*N) group, ^b)^the fresh sample versus Modified (F*M) group, ^c)^the fresh sample vs Kitazato (F*K) group, ^d)^the Nakagata versus Modified (N*M) group, ^e)^the Nakagata versus Kitazato (N*K), ^f)^ Modified versus Kitazato (M*K) group and ^g)^Nakagata versus Modified versus Kitazato (N*M*K).

As seen from the sperm morphology, there was a significant difference before and after vitrification in all of the treatment groups. The modified and Kitazato groups showed significantly better sperm morphology than Nakagata, while Kitazato was not significantly better than the modified. Kitazato was considered the best, even though its average score slightly differed from the Modification group (42% and 41%). In line with the research of Darsini [21], a decrease in normal morphology after sperm cryopreservation was identified in the present research. This research also confirmed the presence of a correlation between morphology and sperm motility, where lower motility could be caused by the irreversible rolling of sperm flagella, thus disrupting the movement of sperm, and even causing immotility. According to Horst, the cut-off value of normal sperm morphology ranged between 67% and 74% [24].

**Figure 1.**
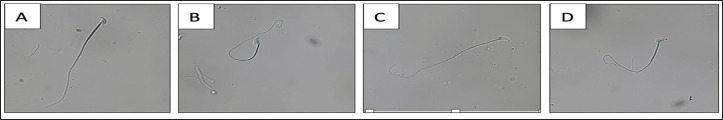
Sperm membrane integrity. (A) Sperm with the damaged plasma membrane, while, (B)–(D) Sperm with intact plasma membrane with variations in the curvature of the sperm’s tail. The observation was performed with 400× magnification.

The sperm membrane integrity exhibited significant differences when comparing pre-vitrification and post-vitrification results in both the Nakagata and modified groups. Significant differences were also observed in the comparisons between the two treatment groups, except in the case of the Modified versus Kitazato comparison. Notably, when assessing all treatment groups collectively, Kitazato demonstrated the highest level of significance, indicating superior sperm membrane integrity. The visual representation of intact and damaged sperm membrane integrity is shown in Figure 1. Moreover, when considering all treatment groups together, Kitazato consistently demonstrated significantly better outcomes. This study underscores the critical importance of maintaining membrane integrity during sperm storage, as the membrane serves as the outermost protective barrier for sperm. Any damage to its function or structure can lead to sperm death, allowing only those with intact membranes to fulfill their fertilization potential [25].

Modified cryoprotectants can protect the integrity of the plasma membrane by 54%. Glycerol in the cryoprotectant modification can protect sperm from damage. Similar results were also found by Sztein [11], who stated that the combination of glycerol and raffinose would be able to protect the mice’s sperm during freezing. He also stated that raffinose in the frost state, like blunt glass, does not mechanically damage the sperm cells. Therefore, repairing the cell plasma membrane will positively impact sperm motility and viability.

**Figure 2.**
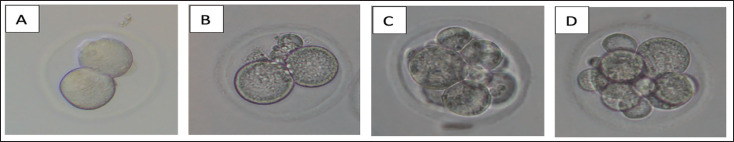
Embryo development on Day 1: (A) good quality, (B) poor quality, and Day-3 (C) good quality, (D) poor quality.

In the context of the CSR parameter, the Nakagata group showed a mean CSR of 42.38% ± 4.74%, the Modification group exhibited a CSR of 54.08% ± 6.73% and the highest CSR was observed in the Kitazato group, with a mean CSR of 65.43% ± 7.29%. Significant differences were found in the CSR values when comparing the Nakagata and Kitazato groups, as well as when considering all treatment groups together (Table 1). The indicator of spermatozoa survival in cryopreservation is determined through CSR, which is calculated based on the ratio of the percentage of total motility in pre-freezing and post-thawing. This CSR measurement has been regarded as a more accurate measurement when compared to total motility, progressive motility, and total motile sperm count in determining the recovery rate of post-thawing spermatozoa [26]. The CSR in this research indicated that the optimum cryoprotectant that produced the highest CSR was the combination of trehalose and glycerol in commercial cryoprotectant packaging.

In contrast, the VR parameter revealed no significant differences between pre-freezing and post-thawing values within all treatment groups or between the two treatment groups. The Nakagata group showed the highest VR percentage, followed by the Kitazato and Modification groups, with no statistically significant differences. The viability rate is an indicator of survival success in cryopreserved sperm. The one-way ANOVA test did not show a significant difference between the three treatment groups in the VR value (*p-*value* =* 0.586). The highest VR value was found in the Nakagata treatment group (69.56% ± 3.92%), but the difference was not significant in other treatment groups (*p-*value > 0.05). The VR value decreased from 72% to 65%–69% after cryopreservation. The VR represents the post-thaw sperm survival rate as measured by sperm viability. It has been widely examined, and results show that viability after thawing is often lower [27].

### The effect of cryoprotectant modification on embryonic development

The progression of embryonic development on Days 1 and 3, categorized as good or poor, is visually represented in Figure 2. Furthermore, Table 2 presents a comprehensive analysis, including statistical comparisons between the fresh and each treatment group. On Day 1, the majority of embryos exhibited favorable development, as indicated by the highest mean value of good embryos in the Kitazato treatment group (58.62% ± 2.52%), followed closely by the modification group (57.46% ± 1.91%) and the Nakagata group (56.35% ± 2.92%). By Day 3, there was a decrease in the number of well-developed embryos compared to Day 1, but the Kitazato group remained superior, with 43.73% ± 5.30% of embryos demonstrating good development. Similar to Day 1, the modification and Nakagata groups exhibited relatively comparable results, with percentages of 40.31% ± 2.94% and 43.01% ± 3.97%, respectively (Table 2).

As seen in Table 2, the average percentage of good embryos in each treatment group decreased after sperm cryopreservation compared to the fresh sample group (control). On day 1, significant differences were found between the fresh and treatment groups, except for Kitazato on day 1. Meanwhile, the differences between Nakagata vs. Modified, Nakagata vs. Kitazato, and Modified vs. Kitazato were insignificant. Based on Day 3 observations, not all embryos developed into a morula. Kitazato was the best, followed by the modified group and the Nakagata group.

**Table 2.** Embryo development quality.

No.	Cleavage rate	Group				*p*-value
		*Fresh* (*n* = 137)	Nakagata (*n *= 257)	Modification (*n* = 204)	Kitazato (*n* = 106)	
1	Day-1 good	64.57 ± 1.55	56.35 ± 2.92	57.46 ± 1.91	58.62 ± 2.52	0.019^a)^ 0.106^e)^ 0.007^b)^ 0.211^f)^ 0.160^c)^ 0.001^g)^ 0.753^d)^
2	Day-3 good	54.38 ± 3.93	43.01 ± 3.97	40.31 ± 2.94	43.73 ± 5.30	0.009^a)^ 0.204^e)^ 0.013^b)^ 0.230^f)^ 0.006^c)^ 0.001^g)^ 0.588^d)^

*Note:* Data are presented as mean ± standard error mean (mean±SEM). ^a)^The fresh sample versus Nakagata (F*N) group, ^b)^the fresh sample versus Modified (F*M) group, ^c)^the fresh sample versus Kitazato (F*K) group, ^d)^the Nakagata versus Modified (N*M) group, ^e)^the Nakagata versus Kitazato (N*K), ^f)^ Modified versus Kitazato (M*K) group, and ^g)^Nakagata versus Modified versus Kitazato (N*M*K).

Based on observations on the first and third days, significant differences between the three types of treatment groups were found (*p-*value 0.001). After conducting LSD post-hoc analysis, it was found that there were significant differences between the Nakagata vs. Kitazato treatment groups (*p-*value 0.001) and Modified versus Kitazato (*p-*value 0.000). On the first and third days of observation, the embryos in the cryoprotectant modification group were not significantly different, but there was a tendency for the mean value to be slightly higher than that of the Nakagata group, whereas the Kitazato group remained the most superior compared to the Nakagata and modified groups. Interestingly, our study diverges from the use of high cryoprotectant concentrations [28]. Although normal sperm morphology decreased after frozen storage, the modified cryoprotectant was able to fertilize oocytes in cIVF due to the presence of glycerol in the modified cryoprotectant. Glycerol has three hydroxyl groups that can bind to proteins left by water when water leaves the cell. Glycerol can diffuse into cells more quickly, change large and sharp ice crystals, and flex the cell membranes, making them stronger [29]. Specifically, we employed a cryoprotectant-modified composition consisting of 10% glycerol and 18% raffinose in this investigation. It was also able to produce 58% of well-developed embryos on day 1 and 40% on day 3. After liquefaction and elimination of the cryoprotectant, the sperm motility of mice reached 59% on raffinose [11]. Although the use of a modified cryoprotectant resulted in lower mouse sperm motility of 40%, it does not inhibit the ability to fertilize oocytes *in vitro*.

In our study, the Kitazato group continued to outperform both the Nakagata and modification groups. The Kitazato kit, a commercially available option comprising glycerol and trehalose, stands as a viable choice for cryoprotectants in the vitrification process, particularly for human sperm, boasting an impressive survival rate of 60% [7]. However, Kitazato does come with certain limitations, including its relatively high cost, difficulty in procurement due to production and transportation delays (attributed to its production in Japan), and extended shipping times that can reduce the medium’s shelf life. In addition, the use of trehalose, as opposed to raffinose, resulted in significantly better sperm cell recovery, with rates of 48% and 36%, respectively.

This research builds upon the findings from our previous review, which suggested that modified cryoprotectants could serve as a viable alternative [30]. This alternative is appealing due to its cost-effectiveness, ease of acquisition, and superior survival rates when compared to other cryoprotectants. The preliminary conclusion drawn from this study rests on the evidence presented, indicating that modified cryoprotectants could indeed serve as an alternative option based on sperm quality and the embryonic development stage. Our research confirms that sperm cryopreservation exerts a negative impact on embryo quality, particularly concerning the cell division stage and cell potency up to the blastocyst stage. In this research, embryonic development beyond the blastocyst stage was not observed. Certain challenges and limitations were encountered in the course of this research. One notable limitation pertains to the suboptimal incubator conditions. During the research period, several other researchers shared the same incubator due to the unavailability of a dedicated incubator for embryo culture. This situation posed challenges, as embryos are highly sensitive and susceptible to environmental factors, including disinfectant fumes such as alcohol, which are commonly used to sterilize equipment before placing them in the incubator.

## Conclusion

This research has demonstrated the potential of modified cryoprotectants as viable alternatives in the context of embryo development. The specific modified composition devised for this study involved the combination of intra and extracellular cryoprotectants, consisting of 10% glycerol and 18% raffinose.

## List of Abbreviations

PRP, Platelet Rich Plasma; PETG, Polyethylene terephthalate glikol; CSR, Cryosurvival Rate; VR, Viability Rate; COC, Cumulus oocyte complex; LSD, Least significant difference; HCG, human chorionic gonadotropin; IVF, in vitro fertilization.
